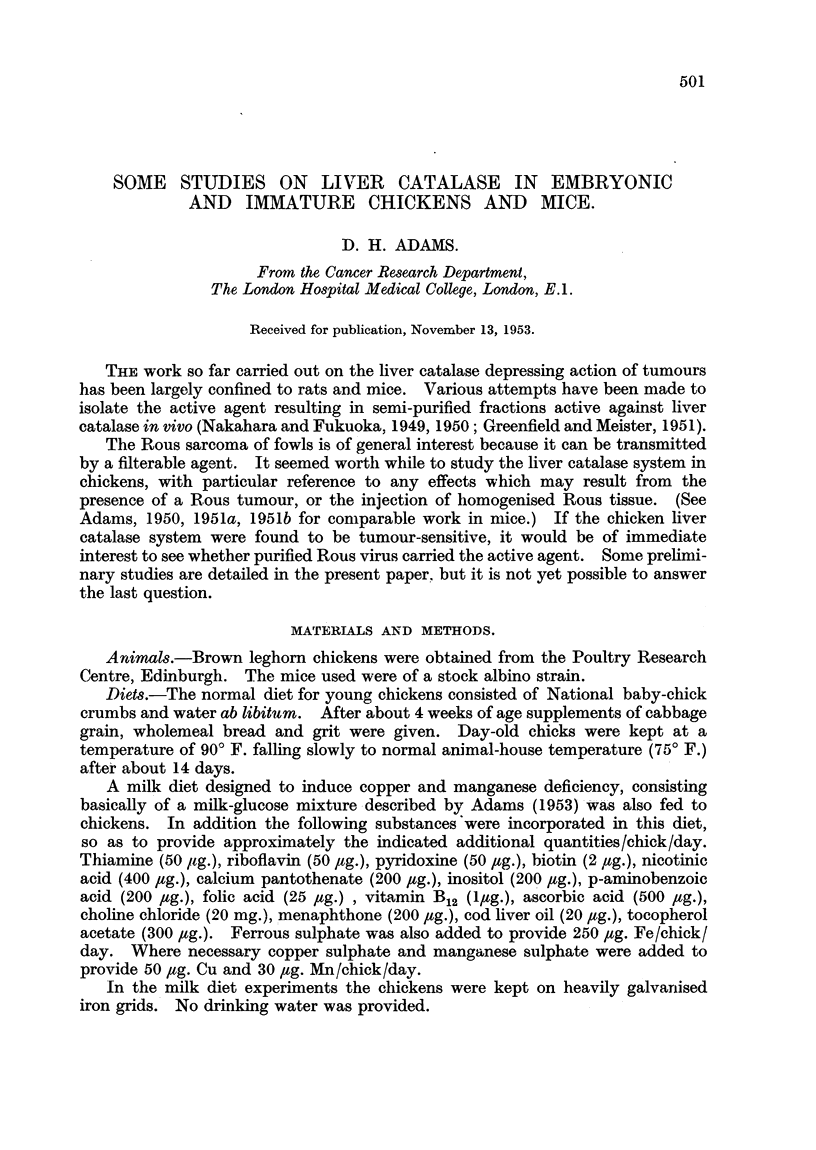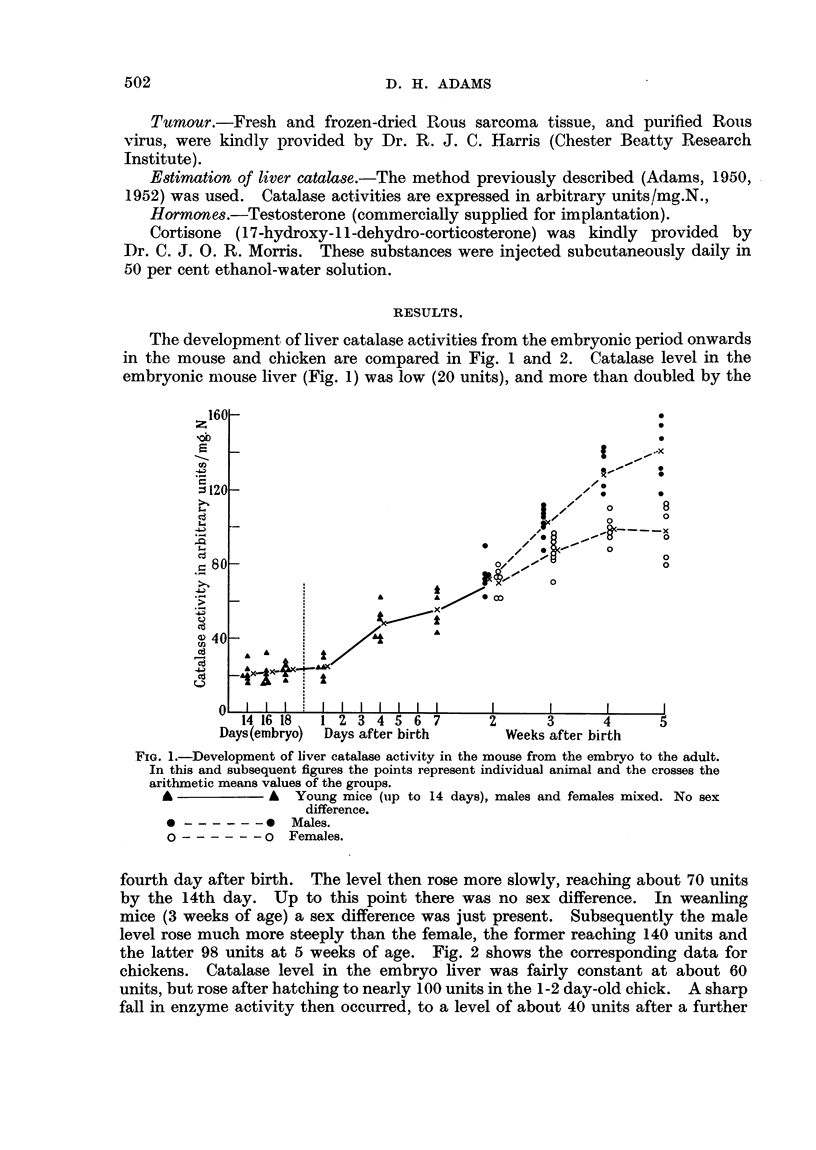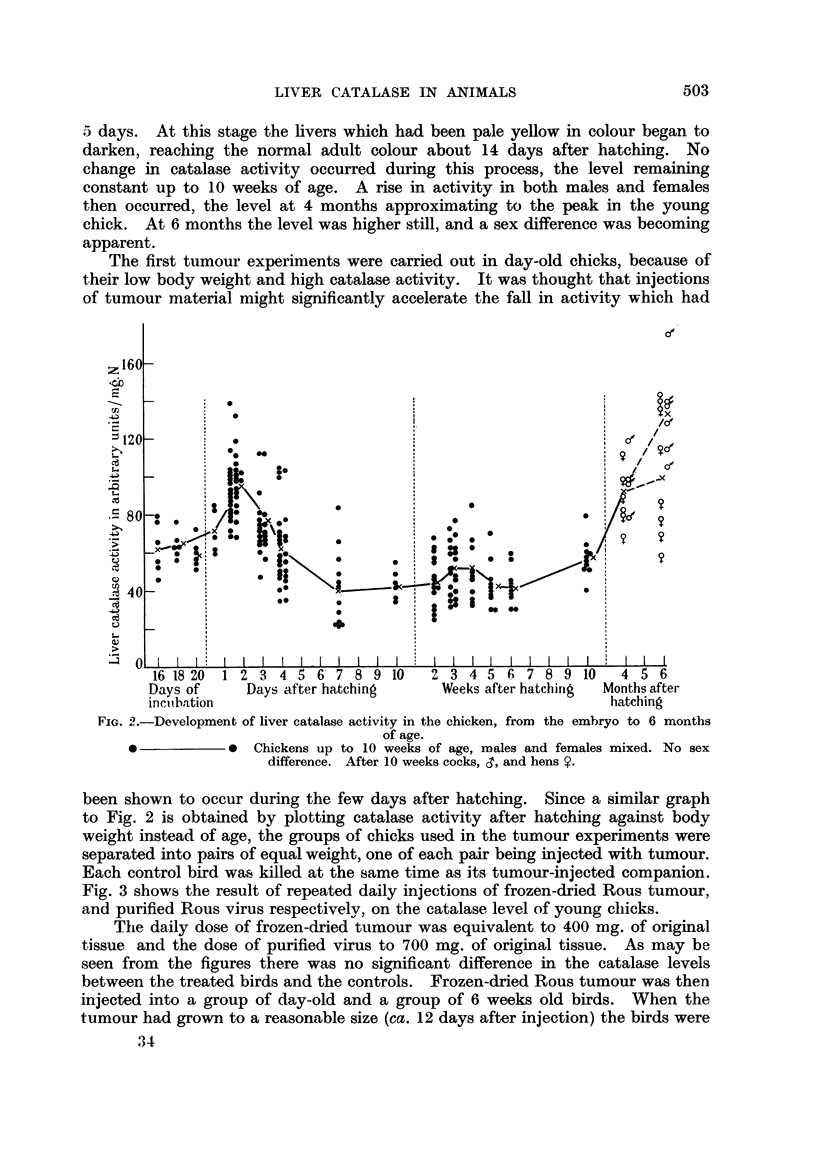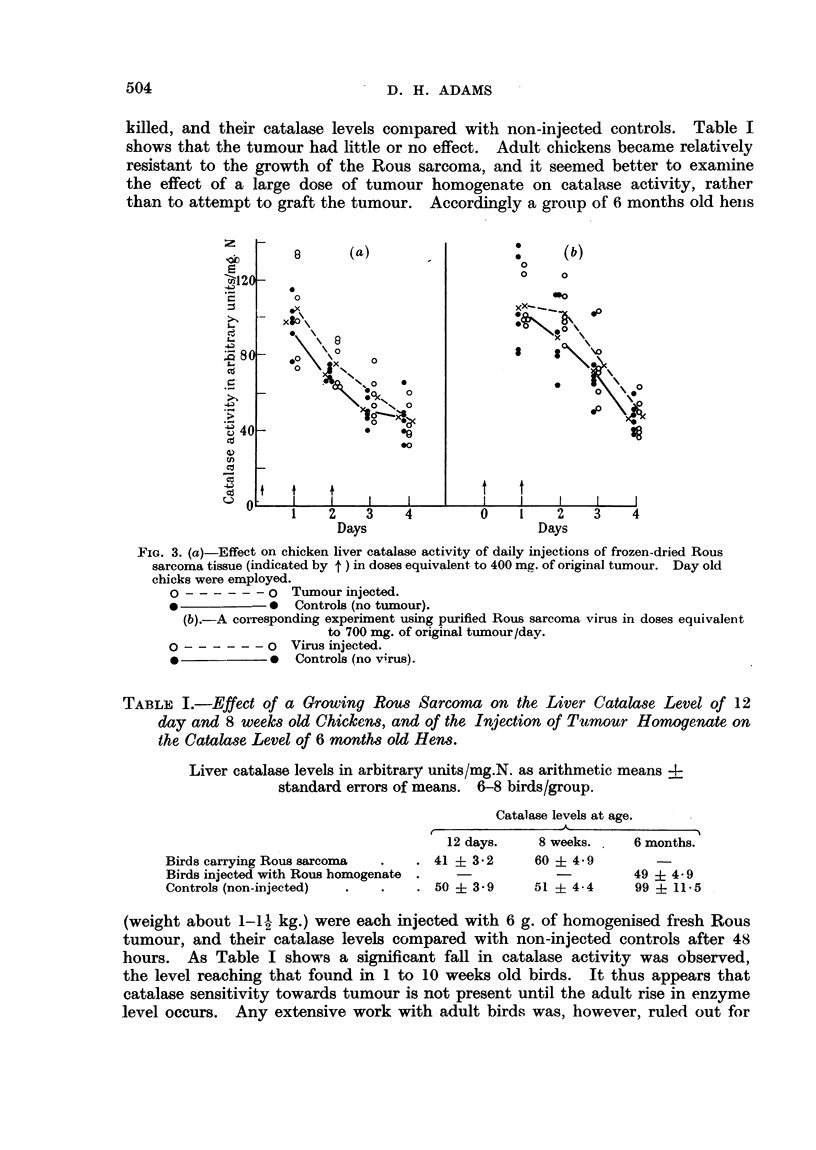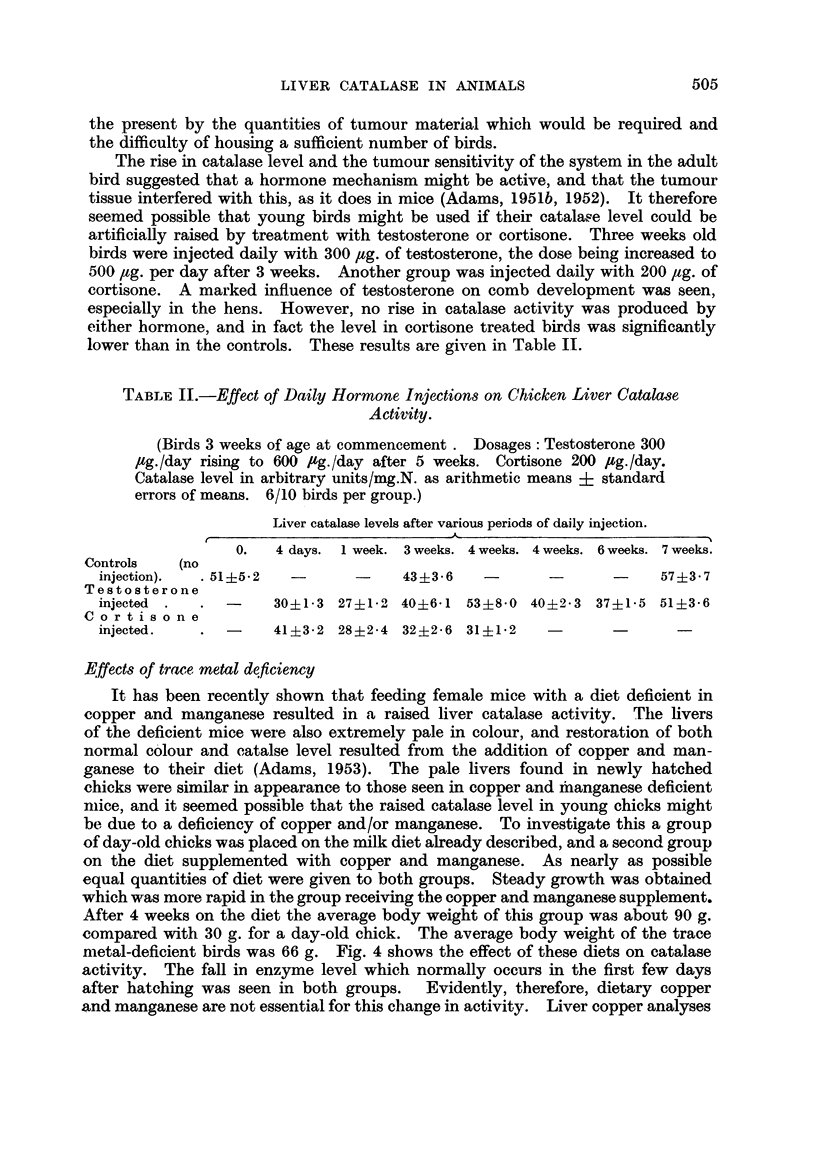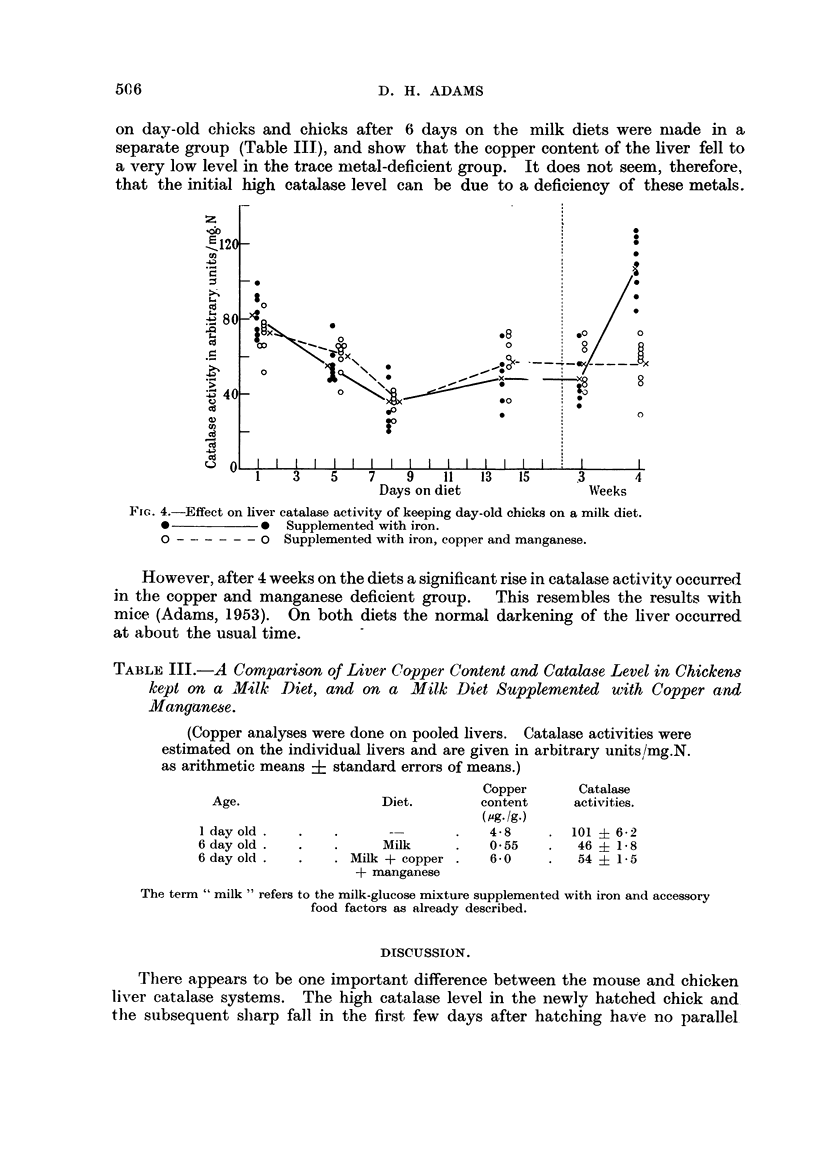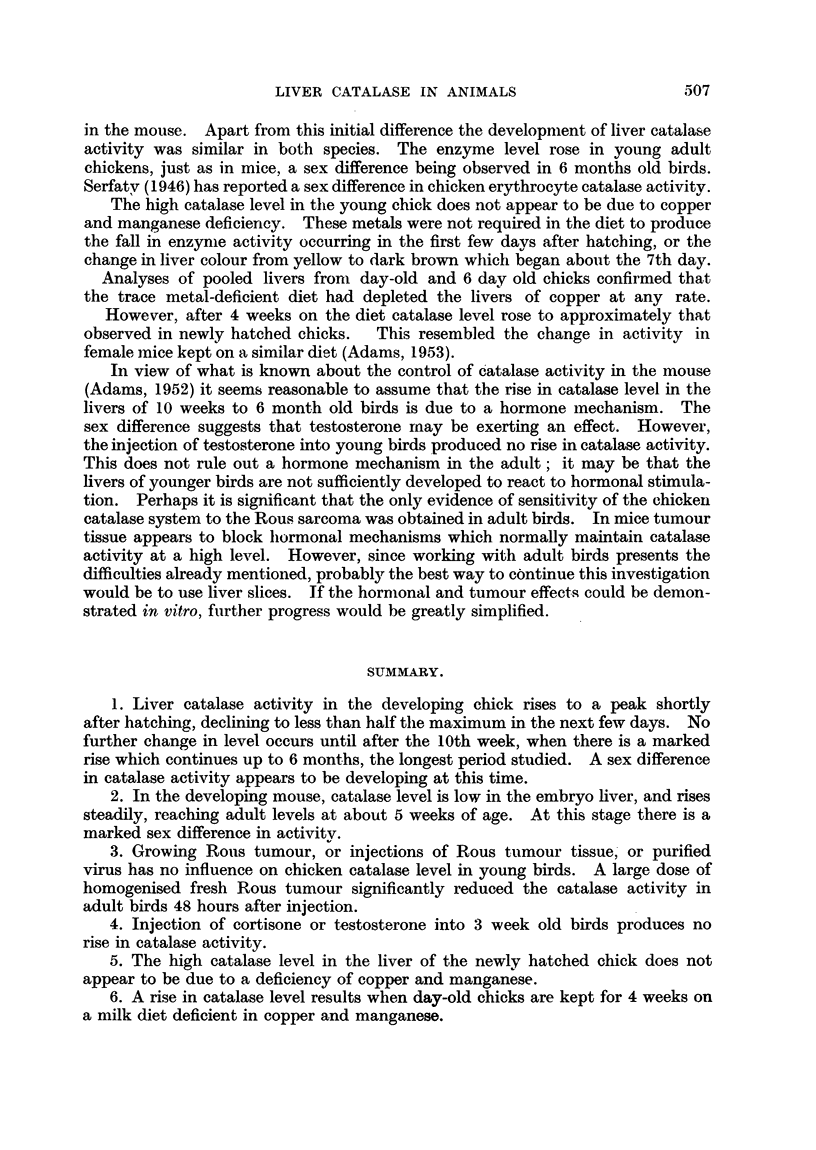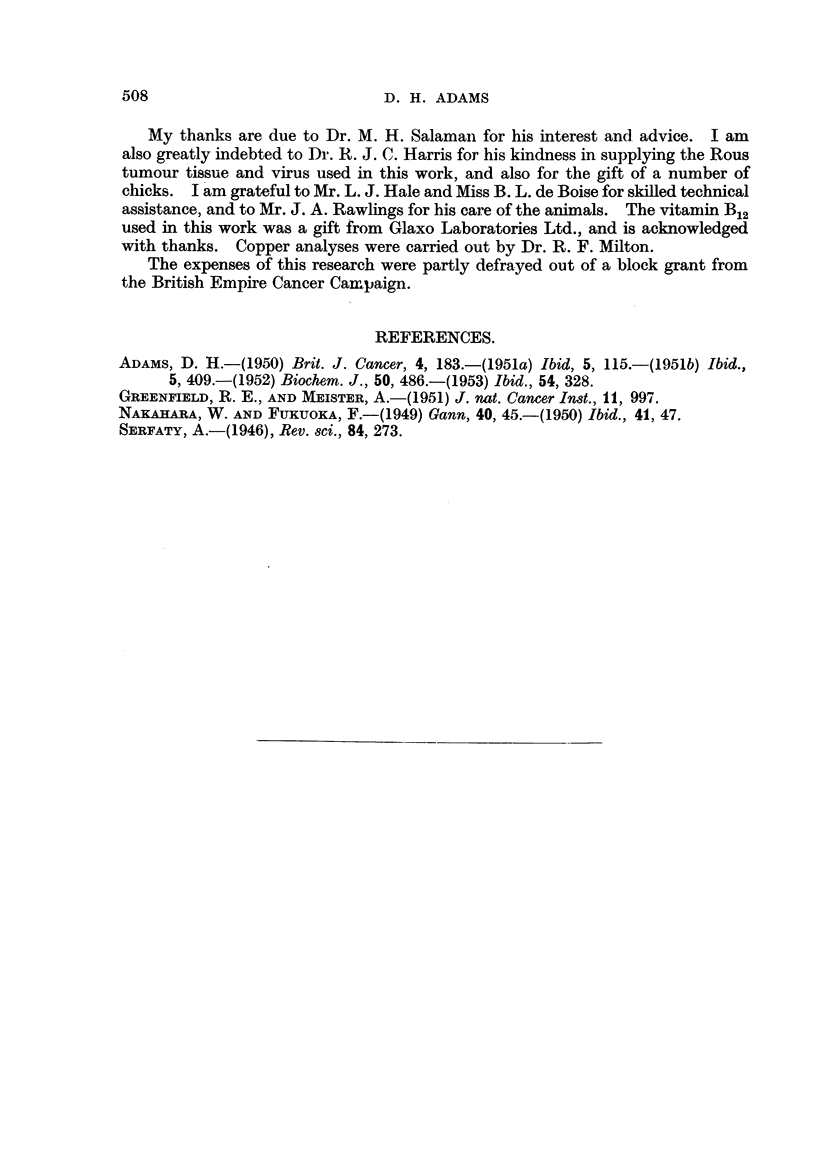# Some Studies on Liver Catalase in Embryonic and Immature Chickens and Mice

**DOI:** 10.1038/bjc.1953.53

**Published:** 1953-12

**Authors:** D. H. Adams


### SOME STUDIES ON LIVER CATALASE IN EMBRYONIC AND IMMATURE CHICKENS AND MICE.

D. H. ADAMS.

*From the Cancer Research Department,*
*The London Hospital Medical College, London, E.1.*

Received for publication, November 13, 1953.

THE work so far carried out on the liver catalase depressing action of tumours has been largely confined to rats and mice. Various attempts have been made to isolate the active agent resulting in semi-purified fractions active against liver catalase *in vivo* (Nakahara and Fukuoka, 1949, 1950 ; Greenfield and Meister, 1951).

The Rous sarcoma of fowls is of general interest because it can be transmitted by a filterable agent. It seemed worth while to study the liver catalase system in chickens, with particular reference to any effects which may result from the presence of a Rous tumour, or the injection of homogenised Rous tissue. (See Adams, 1950, 1951*a*, 1951*b* for comparable work in mice.) If the chicken liver catalase system were found to be tumour-sensitive, it would be of immediate interest to see whether purified Rous virus carried the active agent. Some preliminary studies are detailed in the present paper, but it is not yet possible to answer the last question.

### MATERIALS AND METHODS.

*Animals.*—Brown leghorn chickens were obtained from the Poultry Research Centre, Edinburgh. The mice used were of a stock albino strain.

*Diets.*—The normal diet for young chickens consisted of National baby-chick crumbs and water *ab libitum.* After about 4 weeks of age supplements of cabbage grain, wholemeal bread and grit were given. Day-old chicks were kept at a temperature of 90° F. falling slowly to normal animal-house temperature (75° F.) after about 14 days.

A milk diet designed to induce copper and manganese deficiency, consisting basically of a milk-glucose mixture described by Adams (1953) was also fed to chickens. In addition the following substances were incorporated in this diet, so as to provide approximately the indicated additional quantities/chick/day. Thiamine (50 $\mu$g.), riboflavin (50 $\mu$g.), pyridoxine (50 $\mu$g.), biotin (2 $\mu$g.), nicotinic acid (400 $\mu$g.), calcium pantothenate (200 $\mu$g.), inositol (200 $\mu$g.), p-aminobenzoic acid (200 $\mu$g.), folic acid (25 $\mu$g.) , vitamin $B_{12}$ (1$\mu$g.), ascorbic acid (500 $\mu$g.), choline chloride (20 mg.), menaphthone (200 $\mu$g.), cod liver oil (20 $\mu$g.), tocopherol acetate (300 $\mu$g.). Ferrous sulphate was also added to provide 250 $\mu$g. Fe/chick/ day. Where necessary copper sulphate and manganese sulphate were added to provide 50 $\mu$g. Cu and 30 $\mu$g. Mn/chick/day.

In the milk diet experiments the chickens were kept on heavily galvanised iron grids. No drinking water was provided.

*Tumour*.—Fresh and frozen-dried Rous sarcoma tissue, and purified Rous virus, were kindly provided by Dr. R. J. C. Harris (Chester Beatty Research Institute).

*Estimation of liver catalase*.—The method previously described (Adams, 1950, 1952) was used. Catalase activities are expressed in arbitrary units/mg.N.,

*Hormones*.—Testosterone (commercially supplied for implantation).

Cortisone (17-hydroxy-11-dehydro-corticosterone) was kindly provided by Dr. C. J. O. R. Morris. These substances were injected subcutaneously daily in 50 per cent ethanol-water solution.

RESULTS.

The development of liver catalase activities from the embryonic period onwards in the mouse and chicken are compared in Fig. 1 and 2. Catalase level in the embryonic mouse liver (Fig. 1) was low (20 units), and more than doubled by the

FIG. 1.—Development of liver catalase activity in the mouse from the embryo to the adult. In this and subsequent figures the points represent individual animal and the crosses the arithmetic means values of the groups.

▲ ———— ▲ Young mice (up to 14 days), males and females mixed. No sex difference.

● - - - - ● Males.

○ - - - - ○ Females.

fourth day after birth. The level then rose more slowly, reaching about 70 units by the 14th day. Up to this point there was no sex difference. In weanling mice (3 weeks of age) a sex difference was just present. Subsequently the male level rose much more steeply than the female, the former reaching 140 units and the latter 98 units at 5 weeks of age. Fig. 2 shows the corresponding data for chickens. Catalase level in the embryo liver was fairly constant at about 60 units, but rose after hatching to nearly 100 units in the 1-2 day-old chick. A sharp fall in enzyme activity then occurred, to a level of about 40 units after a further

5 days. At this stage the livers which had been pale yellow in colour began to darken, reaching the normal adult colour about 14 days after hatching. No change in catalase activity occurred during this process, the level remaining constant up to 10 weeks of age. A rise in activity in both males and females then occurred, the level at 4 months approximating to the peak in the young chick. At 6 months the level was higher still, and a sex difference was becoming apparent.

The first tumour experiments were carried out in day-old chicks, because of their low body weight and high catalase activity. It was thought that injections of tumour material might significantly accelerate the fall in activity which had

FIG. 2.—Development of liver catalase activity in the chicken, from the embryo to 6 months of age.

●————● Chickens up to 10 weeks of age, males and females mixed. No sex difference. After 10 weeks cocks, ♂, and hens ♀.

been shown to occur during the few days after hatching. Since a similar graph to Fig. 2 is obtained by plotting catalase activity after hatching against body weight instead of age, the groups of chicks used in the tumour experiments were separated into pairs of equal weight, one of each pair being injected with tumour. Each control bird was killed at the same time as its tumour-injected companion. Fig. 3 shows the result of repeated daily injections of frozen-dried Rous tumour, and purified Rous virus respectively, on the catalase level of young chicks.

The daily dose of frozen-dried tumour was equivalent to 400 mg. of original tissue and the dose of purified virus to 700 mg. of original tissue. As may be seen from the figures there was no significant difference in the catalase levels between the treated birds and the controls. Frozen-dried Rous tumour was then injected into a group of day-old and a group of 6 weeks old birds. When the tumour had grown to a reasonable size (*ca.* 12 days after injection) the birds were killed, and their catalase levels compared with non-injected controls. Table I shows that the tumour had little or no effect. Adult chickens became relatively resistant to the growth of the Rous sarcoma, and it seemed better to examine the effect of a large dose of tumour homogenate on catalase activity, rather than to attempt to graft the tumour. Accordingly a group of 6 months old hens

FIG. 3. (*a*)—Effect on chicken liver catalase activity of daily injections of frozen-dried Rous sarcoma tissue (indicated by ↑) in doses equivalent to 400 mg. of original tumour. Day old chicks were employed.

o – – – – – – – o Tumour injected.
● ——————— ● Controls (no tumour).

(*b*).—A corresponding experiment using purified Rous sarcoma virus in doses equivalent to 700 mg. of original tumour/day.

o – – – – – – – o Virus injected.
● ——————— ● Controls (no virus).

TABLE I.—*Effect of a Growing Rous Sarcoma on the Liver Catalase Level of* 12 *day and* 8 *weeks old Chickens, and of the Injection of Tumour Homogenate on the Catalase Level of* 6 *months old Hens.*

Liver catalase levels in arbitrary units/mg.N. as arithmetic means ± standard errors of means. 6–8 birds/group.

| | Catalase levels at age. | | |
|---|---|---|---|
| | 12 days. | 8 weeks. | 6 months. |
| Birds carrying Rous sarcoma | 41 ± 3·2 | 60 ± 4·9 | — |
| Birds injected with Rous homogenate | — | — | 49 ± 4·9 |
| Controls (non-injected) | 50 ± 3·9 | 51 ± 4·4 | 99 ± 11·5 |

(weight about 1–1½ kg.) were each injected with 6 g. of homogenised fresh Rous tumour, and their catalase levels compared with non-injected controls after 48 hours. As Table I shows a significant fall in catalase activity was observed, the level reaching that found in 1 to 10 weeks old birds. It thus appears that catalase sensitivity towards tumour is not present until the adult rise in enzyme level occurs. Any extensive work with adult birds was, however, ruled out for the present by the quantities of tumour material which would be required and the difficulty of housing a sufficient number of birds.

The rise in catalase level and the tumour sensitivity of the system in the adult bird suggested that a hormone mechanism might be active, and that the tumour tissue interfered with this, as it does in mice (Adams, 1951*b*, 1952). It therefore seemed possible that young birds might be used if their catalase level could be artificially raised by treatment with testosterone or cortisone. Three weeks old birds were injected daily with 300 $\mu$g. of testosterone, the dose being increased to 500 $\mu$g. per day after 3 weeks. Another group was injected daily with 200 $\mu$g. of cortisone. A marked influence of testosterone on comb development was seen, especially in the hens. However, no rise in catalase activity was produced by either hormone, and in fact the level in cortisone treated birds was significantly lower than in the controls. These results are given in Table II.

TABLE II.—*Effect of Daily Hormone Injections on Chicken Liver Catalase Activity.*

(Birds 3 weeks of age at commencement . Dosages : Testosterone 300 $\mu$g./day rising to 600 $\mu$g./day after 5 weeks. Cortisone 200 $\mu$g./day. Catalase level in arbitrary units/mg.N. as arithmetic means $\pm$ standard errors of means. 6/10 birds per group.)

| | Liver catalase levels after various periods of daily injection. | | | | | | | |
|---|---|---|---|---|---|---|---|---|
| | 0. | 4 days. | 1 week. | 3 weeks. | 4 weeks. | 4 weeks. | 6 weeks. | 7 weeks. |
| Controls (no injection). | $51\pm5\cdot2$ | — | — | $43\pm3\cdot6$ | — | — | — | $57\pm3\cdot7$ |
| Testosterone injected | — | $30\pm1\cdot3$ | $27\pm1\cdot2$ | $40\pm6\cdot1$ | $53\pm8\cdot0$ | $40\pm2\cdot3$ | $37\pm1\cdot5$ | $51\pm3\cdot6$ |
| Cortisone injected. | — | $41\pm3\cdot2$ | $28\pm2\cdot4$ | $32\pm2\cdot6$ | $31\pm1\cdot2$ | — | — | — |

*Effects of trace metal deficiency*

It has been recently shown that feeding female mice with a diet deficient in copper and manganese resulted in a raised liver catalase activity. The livers of the deficient mice were also extremely pale in colour, and restoration of both normal colour and catalse level resulted from the addition of copper and manganese to their diet (Adams, 1953). The pale livers found in newly hatched chicks were similar in appearance to those seen in copper and manganese deficient mice, and it seemed possible that the raised catalase level in young chicks might be due to a deficiency of copper and/or manganese. To investigate this a group of day-old chicks was placed on the milk diet already described, and a second group on the diet supplemented with copper and manganese. As nearly as possible equal quantities of diet were given to both groups. Steady growth was obtained which was more rapid in the group receiving the copper and manganese supplement. After 4 weeks on the diet the average body weight of this group was about 90 g. compared with 30 g. for a day-old chick. The average body weight of the trace metal-deficient birds was 66 g. Fig. 4 shows the effect of these diets on catalase activity. The fall in enzyme level which normally occurs in the first few days after hatching was seen in both groups. Evidently, therefore, dietary copper and manganese are not essential for this change in activity. Liver copper analyses on day-old chicks and chicks after 6 days on the milk diets were made in a separate group (Table III), and show that the copper content of the liver fell to a very low level in the trace metal-deficient group. It does not seem, therefore, that the initial high catalase level can be due to a deficiency of these metals.

FIG. 4.—Effect on liver catalase activity of keeping day-old chicks on a milk diet.
● ——————— ● Supplemented with iron.
O - - - - - - - O Supplemented with iron, copper and manganese.

However, after 4 weeks on the diets a significant rise in catalase activity occurred in the copper and manganese deficient group. This resembles the results with mice (Adams, 1953). On both diets the normal darkening of the liver occurred at about the usual time.

TABLE III.—*A Comparison of Liver Copper Content and Catalase Level in Chickens kept on a Milk Diet, and on a Milk Diet Supplemented with Copper and Manganese.*

(Copper analyses were done on pooled livers. Catalase activities were estimated on the individual livers and are given in arbitrary units/mg.N. as arithmetic means ± standard errors of means.)

| Age. | Diet. | Copper content (μg./g.) | Catalase activities. |
|---|---|---|---|
| 1 day old | — | 4·8 | 101 ± 6·2 |
| 6 day old | Milk | 0·55 | 46 ± 1·8 |
| 6 day old | Milk + copper + manganese | 6·0 | 54 ± 1·5 |

The term " milk " refers to the milk-glucose mixture supplemented with iron and accessory food factors as already described.

### DISCUSSION.

There appears to be one important difference between the mouse and chicken liver catalase systems. The high catalase level in the newly hatched chick and the subsequent sharp fall in the first few days after hatching have no parallel in the mouse. Apart from this initial difference the development of liver catalase activity was similar in both species. The enzyme level rose in young adult chickens, just as in mice, a sex difference being observed in 6 months old birds. Serfaty (1946) has reported a sex difference in chicken erythrocyte catalase activity.

The high catalase level in the young chick does not appear to be due to copper and manganese deficiency. These metals were not required in the diet to produce the fall in enzyme activity occurring in the first few days after hatching, or the change in liver colour from yellow to dark brown which began about the 7th day. Analyses of pooled livers from day-old and 6 day old chicks confirmed that the trace metal-deficient diet had depleted the livers of copper at any rate. However, after 4 weeks on the diet catalase level rose to approximately that observed in newly hatched chicks. This resembled the change in activity in female mice kept on a similar diet (Adams, 1953).

In view of what is known about the control of catalase activity in the mouse (Adams, 1952) it seems reasonable to assume that the rise in catalase level in the livers of 10 weeks to 6 month old birds is due to a hormone mechanism. The sex difference suggests that testosterone may be exerting an effect. However, the injection of testosterone into young birds produced no rise in catalase activity. This does not rule out a hormone mechanism in the adult; it may be that the livers of younger birds are not sufficiently developed to react to hormonal stimulation. Perhaps it is significant that the only evidence of sensitivity of the chicken catalase system to the Rous sarcoma was obtained in adult birds. In mice tumour tissue appears to block hormonal mechanisms which normally maintain catalase activity at a high level. However, since working with adult birds presents the difficulties already mentioned, probably the best way to continue this investigation would be to use liver slices. If the hormonal and tumour effects could be demonstrated *in vitro*, further progress would be greatly simplified.

### SUMMARY.

1. Liver catalase activity in the developing chick rises to a peak shortly after hatching, declining to less than half the maximum in the next few days. No further change in level occurs until after the 10th week, when there is a marked rise which continues up to 6 months, the longest period studied. A sex difference in catalase activity appears to be developing at this time.

2. In the developing mouse, catalase level is low in the embryo liver, and rises steadily, reaching adult levels at about 5 weeks of age. At this stage there is a marked sex difference in activity.

3. Growing Rous tumour, or injections of Rous tumour tissue, or purified virus has no influence on chicken catalase level in young birds. A large dose of homogenised fresh Rous tumour significantly reduced the catalase activity in adult birds 48 hours after injection.

4. Injection of cortisone or testosterone into 3 week old birds produces no rise in catalase activity.

5. The high catalase level in the liver of the newly hatched chick does not appear to be due to a deficiency of copper and manganese.

6. A rise in catalase level results when day-old chicks are kept for 4 weeks on a milk diet deficient in copper and manganese.

My thanks are due to Dr. M. H. Salaman for his interest and advice. I am also greatly indebted to Dr. R. J. C. Harris for his kindness in supplying the Rous tumour tissue and virus used in this work, and also for the gift of a number of chicks. I am grateful to Mr. L. J. Hale and Miss B. L. de Boise for skilled technical assistance, and to Mr. J. A. Rawlings for his care of the animals. The vitamin $B_{12}$ used in this work was a gift from Glaxo Laboratories Ltd., and is acknowledged with thanks. Copper analyses were carried out by Dr. R. F. Milton.

The expenses of this research were partly defrayed out of a block grant from the British Empire Cancer Campaign.

### REFERENCES.

ADAMS, D. H.—(1950) *Brit. J. Cancer*, **4**, 183.—(1951*a*) *Ibid*, **5**, 115.—(1951*b*) *Ibid.*, **5**, 409.—(1952) *Biochem. J.*, **50**, 486.—(1953) *Ibid.*, **54**, 328.

GREENFIELD, R. E., AND MEISTER, A.—(1951) *J. nat. Cancer Inst.*, **11**, 997.

NAKAHARA, W. AND FUKUOKA, F.—(1949) *Gann*, **40**, 45.—(1950) *Ibid.*, **41**, 47.

SERFATY, A.—(1946), *Rev. sci.*, **84**, 273.